# What is the role of advanced physiotherapy practice for adults in urgent care and emergency department settings? A scoping review protocol.

**DOI:** 10.12688/hrbopenres.14245.1

**Published:** 2025-10-29

**Authors:** Christina Hayes, Mairead Conneely, Rosa McNamara, Rosie Quinn, Helen French, François Desmeules, Ruth Kilcawley, Karen McCreesh, Rose Galvin

**Affiliations:** 1 School of Allied Health, Ageing Research Centre, Health Research Institute,, University of Limerick Faculty of Education and Health Sciences, Limerick, County Limerick, Ireland; 2 Clinical Lead, National Emergency Medicine Programme, St Vincent's Hospital, Dublin, Ireland; 3 Our Lady of Lourdes Hospital, Drogheda, County Dublin, Ireland; 4 Royal College of Surgeons in Ireland Faculty of Medicine and Health Sciences, Dublin, Leinster, Ireland; 5 5School of Rehabilitation, Faculty of Medicine, Université de Montréal, Montreal, QC, Canada; 6 Hôpital Maisonneuve-Rosemont Research Center, Université de Montréal Affiliated Research Center, Montreal, QC, Canada; 7 National Health & Social Care Professions Office, Health Service Executive, St Mary's Campus, County Dublin, Ireland

**Keywords:** Advanced Practice Physiotherapy, APP, extended-scope physiotherapy, extended-scope, advanced practice, primary contact physiotherapy, first-contact physiotherapy, extended scope of practice.

## Abstract

**Introduction:**

The Advanced Practice Physiotherapy (APP)bmodel of care is implemented internationally, with notable variations in its application across different healthcare systems. Nonetheless, APP has been shown as a means of mitigating against emergency department (ED) crowding and reducing pressure on EDs. This protocol outlines the methods in conducting a scoping review to identify the extent of APP roles in urgent care and ED settings, their expertise levels, the profile of patients they treat and the components of care they provide.

**Methods:**

This scoping review will be conducted in line with the Joanna Briggs Institute framework for scoping reviews. The Preferred Reporting Items for Systematic Reviews and Meta-analysis extension for scoping reviews (PRISMA-ScR) will be used to guide the reporting of this review. This review will include published studies of any design that are focussed on APP assessment and/ or subsequent treatment in the ED or urgent care setting among adults. The electronic databases: Medline (Ovid), Pubmed, CINAHL Complete, EMBASE, Epistemonikos, Central Register of Controlled Trials in the Cochrane Library and Scopus, trial registries and grey literature databases will be searched. The reference list of included sources of evidence in the review will be searched for additional sources. The methodological quality of the studies will not be formally explored. Relevant data will be extracted from each article using a predefined data extraction form by two independent reviewers. Data synthesis will be derived from the ‘PCC’ framework (population, concept, and context).

**Conclusions:**

This scoping review will serve to characterise the roles, responsibilities, levels of expertise and the specific components of care that APP offers in urgent and emergency care settings.

## Introduction

Emergency Departments (ED) are under increasing pressure internationally ^1, 2^. In Ireland, 1.29 million ED presentations were reported in 2021 ^3^, with an increase to 1.36 million in 2023 ^4^, where attendees spent an average of 12 hours in the ED ^5^. Global efforts have been made to mitigate against ED overcrowding and improve healthcare efficiency, costs and patient safety ^6^. One such method of reform has been the implementation of advanced practice physiotherapy (APP) within the ED setting internationally ^7– 9^. These new models of care in which allied health professionals, including physiotherapists, have emerged as key health providers in the ED, especially given that more than 25% of all ED presentations are people with musculoskeletal disorders ^7^.

An international consensus regarding a common understanding of the definition of APP is lacking ^10^. The World Physiotherapy organisation described APP as a higher level of physiotherapy practice requiring advanced clinical skills, knowledge and autonomy to improve patient outcomes and provide clinical leadership, typically practiced by a small recognised group of experts in the field ^10^. Moreover, in certain jurisdictions, APPs face restrictions on the services they are permitted to offer and are not acknowledged as salaried clinical grades ^11^.

APP is still in its early stages of development, with variability in its implementation in the ED and urgent care setting depending on the country and professional regulations. APP practitioners’ scope of practice may also vary within the same jurisdiction ^7^. For instance, common APP practices in the ED may include the autonomous care of patients. This may involve making diagnoses, requesting medical imaging, prescription of medications and independent discharge planning for patients in the ED ^7, 12– 14^. This contrasts to usual physiotherapy practice wherein patients are typically referred after a physician’s initial assessment and initiation of treatment ^7^.

Ireland, like many other jurisdictions, lacks formal recognition or salaried clinical grading for APP. APP has however, demonstrated reduced length of stay ^15^, reduced wait time ^8, 15^ and high levels of satisfaction in the ED ^16^. Furthermore, APP is seen as a model of care with the potential to address some of the most pressing healthcare challenges internationally including lack of access to primary care services, escalating wait times for patients in urgent care settings and a shortage of healthcare professionals due to burnout and stress ^17, 18^. The implementation of models of care that maximise the scope of practice of healthcare professionals, including physiotherapists, have a positive impact at the patient, community and healthcare system levels ^17^.

Evidence examining the effectiveness of physiotherapy in the ED has previously been synthesised ^7, 12^. A previous scoping review of 27 studies that examined the evidence in relation to physiotherapists’ roles in the ED reported a lack of robust research to inform policy ^12^. Furthermore, this scoping review explored the role of physiotherapists as a whole, not specific to APP practitioners ^12^. Another systematic review by Desmeules and colleagues conducted a systematic review that explored physiotherapist care for patients with musculoskeletal disorders in the ED ^7^. Results from this review demonstrated that physiotherapy roles vary depending on the setting and legalisation and reported that a limited number of high quality studies were identified for inclusion ^7^. Given that both reviews explored the role of physiotherapists as a whole in the ED, not specific to APP practitioner roles, and the recent global shift towards APP care in the ED, an update of the available evidence for APP roles in the ED is warranted.

A scoping review provides explicit methods to comprehensively summarise evidence consisting of variable study designs with the objective of informing policy, practice and inform future research ^19^.

The overall aim of this scoping review is to explore the role of APP practitioners in urgent care and emergency department settings. Specifically, this study will aim to:

- Characterise the role, responsibilities, levels of expertise, and components of care that APP offers; and
- Describe profile of patients that are treated by APP practitioners in urgent and emergency care settings.

This information will be used to inform a foundational concept of APP in urgent and emergency care settings and inform the future implementation of APP policy, practice and research.

## Methods

This scoping review will be conducted in line with the Joanna Briggs Institute (JBI) methodology for scoping reviews ^20^. The scoping review framework proposed by Arksey and O’Malley will be employed ^21^. The report will be guided by the Preferred Reporting Items for Systematic Reviews and Meta-Analyses Extension for Scoping Reviews (PRISMA-ScR) ^22^. This protocol is reported in line with the PRISMA-P checklist ^23^, and registered on the Open Science Framework: https://doi.org/10.17605/OSF.IO/W4FXH.

### Ethics

Formal ethical approval is not required for this review as all data included are anonymous secondary data.

### Eligibility criteria

The ‘PCC’ mnemonic (population, concept, and context) derived by Peters and colleagues was adopted to guide the eligibility criteria for this scoping review ^20^.

***Population***

Adults (aged ≥18 years) who present to the ED or urgent care unit (e.g. local injury unit).

***Concept***

The intervention of interest is APP assessment and/or treatment in the ED urgent care setting. The assessment/intervention may be conducted independently or as part of a multidisciplinary (MDT) plan of care. The following definition of APP that was derived from the findings of international focus groups on the global perspectives of APP along with the World Physiotherapy definition ^10^ will be used:

“Expert physiotherapists who employ a higher level of competencies and expertise with additional responsibilities and autonomy to manage complex and challenging health needs of individuals, families and populations within or beyond their scope of practice. Advanced practice physiotherapists demonstrate competencies as expert clinician, communicator, collaborator, leader, health advocate, scholar, and professional” ^17^.

***Context***

Published studies of any design that are focussed on APP assessment and/ or subsequent treatment in the ED or urgent care setting will be considered eligible. There will be no date or language restrictions applied to the database search.

**Inclusion criteria**

- Review articles including systematic reviews, scoping reviews, and rapid reviews; quantitative studies (observational and experimental); qualitative and mixed method studies; clinical care guidelines; and grey literature.
- Grey literature including studies, reports and published articles that focus on APP assessment and/or subsequent treatment among adults in the ED or urgent care setting.

**Exclusion criteria**

- Articles in which APP is initiated on a hospital ward and not in the ED;
- Conference abstracts;
- Primary care based studies.

### Search strategy

The search strategy for this Scoping review will be developed in collaboration with an information specialist at the University of Limerick (UL). An initial limited search of the CINAHL via EBSCO and OVID MEDLINE databases will be conducted to identify articles on the topic using the search strategy outlined in the ‘Extended data’. A logic grid that uses Boolean operators will structure searches. The text words contained in the titles and abstracts of relevant articles, and the index terms/ key words used to describe the articles will inform subject headings for a full search strategy with the input of a specialist health sciences librarian at UL.

A comprehensive search will be completed by MC in the following electronic databases: Medline (Ovid), Pubmed, EMBASE, CINAHL Complete, Epistemonikos, Central Register of Controlled Trials in the Cochrane Library and Scopus. Trial registries including the WHO International Clinical Trials Registry Platform (ICTRP): www.who.int/ictrp/en/ will be searched. A grey literature search will be conducted in the following databases: grey literature sources ( DART-Europe E-theses portal, Open Grey, and Trip Medical database). The reference list of included sources of evidence in the review will be searched for additional sources via backward and forward citation searching by CH and MC.

### Study selection

Study selection will be completed using a two-step process. Firstly, studies will be downloaded to Endnote, and duplicates removed. The screening process will be carried out using Rayyan screening software. Titles and abstracts will be screened by two independent review authors (CH and MC). Step 2 will involve two independent review authors (CH and MC) screening full-text studies according to the inclusion criteria, which will be outlined in a Preferred Reporting Items for Systematic Reviews and Meta-analyses extension for scoping review (PRISMA-ScR) flow diagram. Any inconsistencies in the inclusion process will be resolved via consensus or third author consultation (RG).

### Charting the data

An adapted standardised data charting form developed by the JBI methodology guidance for scoping reviews will be used to guide the data extraction process ( Table 1) ^20^. Authors of papers will be contacted to request missing or additional data, where required. The data charting form will be piloted on two studies by two independent authors (CH and MC) to ensure that all necessary data will be captured appropriately. The draft data charting form ( Table 1) may be refined at review stage as required. Data will be independently charted by one researcher (CH) and cross-checked against original articles by a second researcher (MC) to ensure the validity of extracted data.

**Table 1.** Adapted data charting form from Joanna Briggs Institute (JBI) methodology for scoping reviews ^20^.

**Scoping Review Details**	
Scoping Review title and publication type:	
Review objective/s:	
Review question/s:	
**Inclusion/Exclusion Criteria**	
Population	
Concept	
Context (e.g. Emergency Department or local injury unit)	
Types of evidence source	
**Evidence source Details and Characteristics**	
Citation details (e.g. author/s, date, title, journal, volume, issue, pages)	
Country	
Context	
Participants (details e.g. age/sex and number)	
**Details/Results extracted from source of evidence** (in relation to the concept of the scoping review)	
Roles of APP	
Training levels of physiotherapists	
Outcomes of interest evaluated in included studies	

Assessment of the risk of bias or methodological quality of included studies is not relevant as the aim of this scoping review is to describe the current APP practices in the ED or urgent care setting ^20^.

### Data analysis and presentation

Data synthesis will be derived from the ‘PCC’ framework (population, concept, and context) by Peters and colleagues ^20^. This will include information on the profile of patients that APP practitioners are treating, intervention components, APP roles and responsibilities and outcomes of interest outlined in included studies. Quantitative data analysis including descriptive analysis, such as, year, country, setting, participant characteristics and outcomes of interest will be conducted using SPSS Version 24 software and presented in a tabular format. Qualitative data will be presented narratively, featuring a summary of the findings in included studies. Basic coding of the qualitative findings will be conducted by two independent reviewers (CH and RG) using NVivo software to identify and categorise the described features of APP according to the population, concept, and context. 

## Study status

This protocol was registered prospectively on the Open Science Framework.

## Discussion

This scoping review protocol describes a systematic approach to investigate the role of APP in urgent care and ED settings. It aims to delineate the extent of their responsibilities, levels of expertise, patient demographics and the specific components of care APP offers. Previous evidence suggests that care provided by physiotherapists in the ED may contribute to shorter waiting times among patients with lower urgency ^12^. However, findings from randomised controlled trials report no notable differences in the effectiveness or costs associated with care delivered by physiotherapists in the ED in comparison to other ED staff ^12^. Given the heterogeneity of APP roles internationally, this scoping review will be used to inform a foundational concept of APP in urgent care and ED settings and enable the future examination of APP pathways, practice and policy.

## Dissemination

The review will abide by the (PRISMA-ScR) ^22^ standardised reporting guidelines and will be published in a peer-reviewed journal and disseminated through discussion with the National Emergency Medicine Programme in Ireland. Scientific outputs will be disseminated in the form of presentations at relevant physiotherapy and emergency medicine conferences.

## Data Availability

No data are associated with this article. Open Science Framework: What is the role of advanced physiotherapy practice for adults in urgent care and emergency department settings? A scoping review protocol. https://doi.org/10.17605/OSF.IO/W4FXH ^24^. This Project contains the following extended data: PRISMA-P checklist Database search terms Open Science Framework: PRISMA-P checklist ^23^ for What is the role of advanced physiotherapy practice for adults in urgent care and emergency department settings? A scoping review protocol. https://doi.org/10.17605/OSF.IO/W4FXH ^24^. Data are available under the terms of the CC0 open licence.
