# Supplementary material for: What is the role of advanced physiotherapy practice for adults in urgent care and emergency department settings? A scoping review protocol
Source: HRB Open Res. 2025 Oct 29;8:116. [Version 1] doi: 10.12688/hrbopenres.14245.1 (PMC12614314; doi:10.12688/hrbopenres.14245.1)
Supplement: Peer review history

# Reviewer report: Reviewer response for version 1

**DOI:** 10.21956/hrbopenres.15667.r51132

**Published:** 2025-11-08

**Authors:** Kaitlyn Maddigan

**Affiliations:** 1 Western University, London, Ontario, Canada

I thank the authors for the opportunity to review their paper. This article is a protocol for a scoping review. The study aims to characterize the role, responsibilities, levels of expertise, and components of care that APP offers; and describe the profile of patients that are treated by APP practitioners in urgent and emergency care settings.

 This study is very interesting and undeniably useful regarding the current state of emergency departments internationally and the potential implications of widespread implementation of APP in these settings. The methodology used is appropriate, although the analysis methods lacks precision. Overall, the protocol is written well.

 Below are some specific comments to take into consideration:

 1) in the introduction, on the final line of the third paragraph you have written: *This contrasts to usual physiotherapy practice wherein patients are typically referred after a physician’s initial assessment and initiation of treatment.*

 - this is not true for all jurisdictions internationally; for example physiotherapists in Canada are primary healthcare practitioners, patients do not require a physician assessment or referral to see a physiotherapist for assessment and diagnosis. 

 2) in the introduction, in the 5th paragraph you have written: *Another systematic review by Desmeules and colleagues conducted a systematic review that explored physiotherapist care for patients with musculoskeletal disorders in the ED.*

 - these is an typo here 'systematic review' has been included twice in this sentence. 

 3) You have described in your objectives that you aim to characterize 'the level of expertise' of the APPs but you have not adequately described what this means or how you plan to do this in the methods. Of all the constructs that you aim to characterize it is the only one with some level of ambiguity. Given that you are referring to 'a level', it could imply that there may be some way to measure this? Will characterizing this be based on the complexity of the cases the APPs handle or some other measurement tool? I think this requires further description/definition to help the reader understand what exactly will contribute to this characterization.

Is the study design appropriate for the research question?

Yes

Is the rationale for, and objectives of, the study clearly described?

Yes

Are sufficient details of the methods provided to allow replication by others?

Partly

Are the datasets clearly presented in a useable and accessible format?

Not applicable

Reviewer Expertise:

My area of research centres around investigating the optimal educational pathway to APP for the physiotherapist, both internationally and in a Canadian context.

I confirm that I have read this submission and believe that I have an appropriate level of expertise to confirm that it is of an acceptable scientific standard.

# Reviewer report: Reviewer response for version 1

**DOI:** 10.21956/hrbopenres.15667.r51135

**Published:** 2025-11-04

**Authors:** Simon P Vella

**Affiliations:** 1 The University of Sydney, Sydney, Australia

Summary

 Thank you for the opportunity to review this exciting project.

 This scoping review protocol aims to explore the role of advanced physiotherapy practice for adults in urgent care and emergency departments, regardless of their complaint. The protocol will use appropriate methodology, such as the JBI framework and PRISMA-ext for Scoping reviews, to answer this important research question.

 The protocol is well written, in which I only have a few comments:

 Line 1 abstract: The Advanced Practice Physiotherapy (APP)bmodel of care is implemented internationally, with notable variations in its application across different healthcare systems.

 Grammar – ‘bmodel of care’, remove *b. *Should read ‘model of care’

 Introduction:

 Paragraph 1: Global efforts have been made to mitigate against ED overcrowding and improve healthcare efficiency, costs and patient safety ^6^.

 Could this be written more simply – Global efforts have been made to *reduce* ED overcrowding and improve healthcare efficiency, costs and patient safety.

 Comment:

 Is emergency department and urgent care setting referring to one (i.e. the same) setting or is this referring to two different settings? That is, Emergency department is one setting and urgent care is another setting.

 For example, in Australia, we have the emergency department which is tied to a hospital. This is one service. We also have urgent care services (i.e. Urgent care setting) which are community based pre-hospital services. This is a different service to the ED. Therefore, the role of the APP in Australia might differ between these two settings as one is a community setting and the other is in the emergency health care setting.

 It might be appropriate to define ‘ *urgent care and emergency department* setting’ in the introduction to provide the reader context of the setting in Ireland, as this may be different to other countries, like Australia.

 Thanks again for the opportunity to review the protocol and I look forward to reading the completed project.

Is the study design appropriate for the research question?

Yes

Is the rationale for, and objectives of, the study clearly described?

Partly

Are sufficient details of the methods provided to allow replication by others?

Yes

Are the datasets clearly presented in a useable and accessible format?

Yes

Reviewer Expertise:

Emergency health services, emergency department, back pain, allied health care

I confirm that I have read this submission and believe that I have an appropriate level of expertise to confirm that it is of an acceptable scientific standard.
